# Spilled Oils: Static Mixtures or Dynamic Weathering and Bioavailability?

**DOI:** 10.1371/journal.pone.0134448

**Published:** 2015-09-02

**Authors:** Mark G. Carls, Marie L. Larsen, Larry G. Holland

**Affiliations:** Auke Bay Laboratories, NMFS, NOAA, Juneau, Alaska, United States of America; University of California, Merced, UNITED STATES

## Abstract

Polynuclear aromatic hydrocarbons (PAHs) from sequestered MV *Selendang Ayu* oil were biologically available in 2008, 3.6 y after it was spilled along Unalaska Island, Alaska. Thermodynamically driven weathering was the most probable mechanism of organism exposure to PAHs. Alkane and PAH composition in oil changed over time as smaller constituents were preferentially lost, indicative of weathering. In contrast, composition of the largest compounds (biomarkers) including triterpanes, hopanes, and steranes remained unchanged. Smaller molecules (the PAHs) lost from stranded oil were observed in indigenous mussels and passive samplers deployed in July 2008. Concentration and composition of PAHs were significantly different than in a non-oiled reference area and patterns observed in mussels were repeated in passive samplers deployed in three zones (intertidal, subtidal, and water). Thus, hydrocarbons lost from one compartment (sequestered whole oil) were detectable in another (mussels and passive samplers) implying aqueous transfer. Quantities of mobile oil constituents were small, yielding uptake concentrations that are likely inconsequential for mussels, but the sensitivity provided by bioaccumulation and passive sampler uptake ensured that dissolved hydrocarbons were detectable.

## Introduction

On 8 December 2004, the MV *Selendang Ayu* (*S*. *Ayu*) ran aground and broke in half in rough seas off Unalaska Island, Alaska (53°38' N, 167°07' W). An estimated 1.34 × 10^6^ L of oil [1.28 × 10^6^ L of intermediate fuel oil (IFO) 380 and 5.56 × 10^4^ L of marine diesel and miscellaneous oils] were discharged. Two types of IFO oil were present, distinguishable by sulfur content among other attributes; these are designated IFO light (IFO_L_, a low sulfur oil) and IFO heavy (IFO_H_, a high sulfur oil). More than 100 km of remote coastline on Unalaska Island were oiled, creating both short- and long-term biological impacts, such as the death of thousands of oiled birds at the time of the spill and biochemical evidence of continued oil exposure through 2008 in harlequin ducks [1–3] and declining PAH concentrations in indigenous mussels [4].

The oil weathering process, driven thermodynamically [5], is important because it is a mechanism for organism exposure to hydrocarbons via aqueous transfer [6–9]. The rate at which polynuclear aromatic hydrocarbons (PAHs) move from stranded whole oil into water is dependent on molecular weight; smaller, less-substituted molecules are lost most rapidly, consistent with first order loss rate kinetics [5]. This mechanism explains the characteristic changes in composition as oil weathers and is well documented [5, 10–13]. Molecules that leave oil enter air or water and subsequently can enter living tissue [14]. Because oil constituents are highly insoluble in water and highly soluble in lipids, living organisms (and passive samplers) typically concentrate low aqueous hydrocarbon levels by a factor of 1000 or more, again a pattern driven by thermodynamics [14]. Thus, stranded oil sets up a mechanism for biological contamination even in the absence of direct contact and this is detectable at very low concentrations. Furthermore, PAHs are toxic [15, 16], often at low concentrations [17–19] therefore biological contamination resultant from weathering processes may have negative consequences for a diversity of aquatic life.

The objectives of this study were to document the source of oil on Unalaska beaches, identify stable and weathering oil constituents, and seek evidence of biologically available oil in indigenous mussels and passive samplers. The presence of two distinct oils provided an opportunity to distinguish weathering processes in stranded oil from an alternative possibility, inert, static mixtures of the primary oils spilled by the *S*. *Ayu*.

Our approach to understanding the implications of residual *S*. *Ayu* oil (SAO) was to demonstrate the presence of stranded oil, identify its source by pattern-matching modeling, determine if it was weathering [5], and link this information to evidence of biological availability (PAH concentration and composition) in indigenous mussels and passive samplers. Direct identification of source oil in organisms and passive samplers can be difficult or impossible because composition changes as oil constituents move from oil to water to organism and because sequestered molecules are subsequently metabolized by enzymes (such as cytochrome P450). Thus, source identification often must be indirect in biological samples and passive samplers. Nonetheless, sufficient source evidence often remains, enough to distinguish petrogenic and pyrogenic sources. Additional evidence for the source of contamination in tissue requires knowledge of the distribution of stranded oil, and this was accomplished visually, chemically, and by reference to previously mapped distributions. The environmental stability of large, multi-ring compounds known as biomarkers (triterpanes, hopanes, and steranes) in SAO, particularly hopanes, allowed definitive pattern-matching modeling that confirmed the origin of lingering oil. Thus, the data demonstrated the largest molecules (biomarkers) remained in stranded oil as static mixtures, whereas smaller molecules (the PAHs) weathered and were biologically available.

## Methods

**Fig 1:**
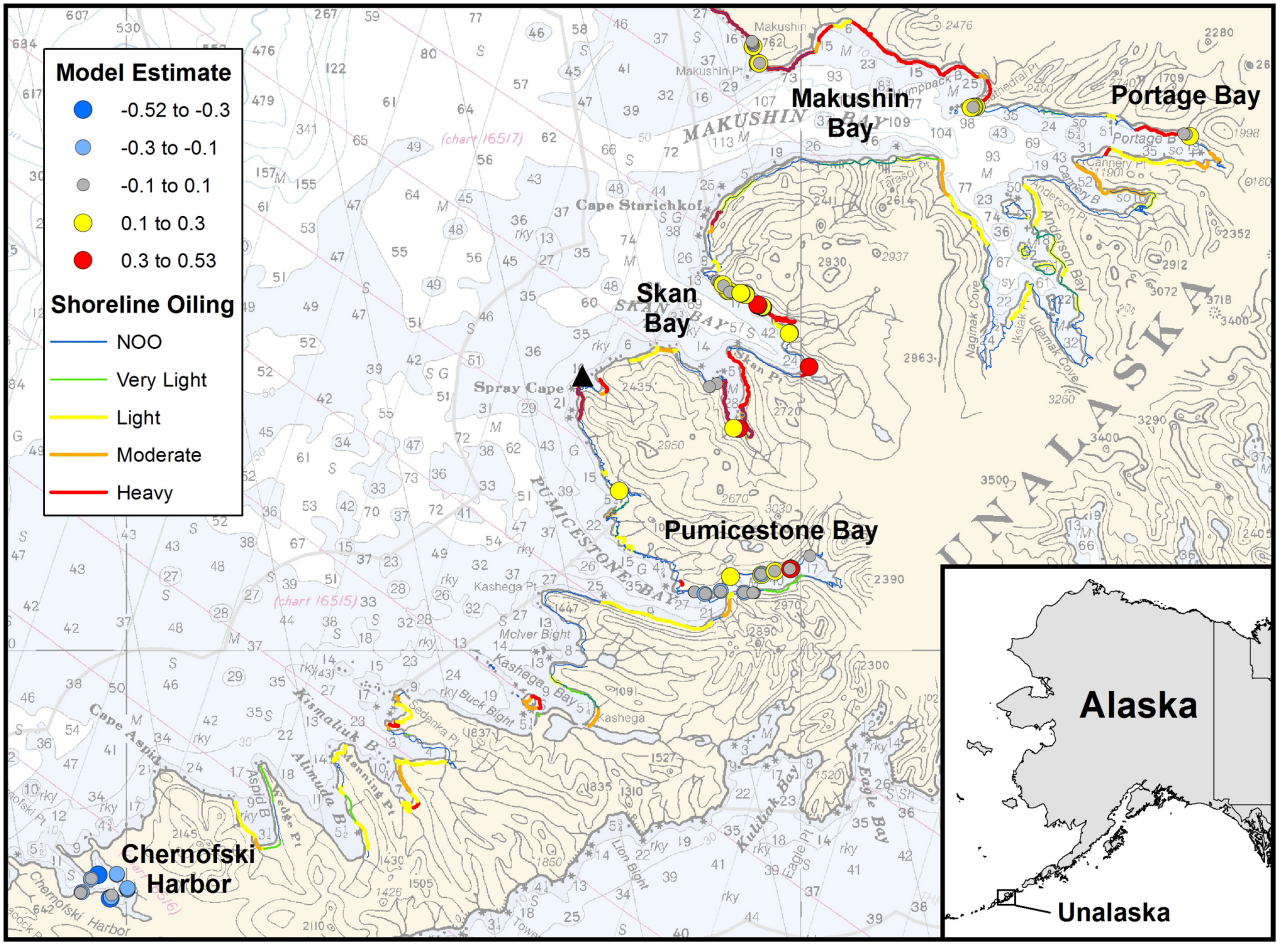
PAH model results for mussel tissue (2008) within the previous *S*. *Ayu* spill area, historically contaminated (Chernofski Harbor), and reference areas (Pumicestone Bay). Shoreline oiling was assessed in 2005. The black triangle marks the location of the wreck. The basemap is NOAA chart 16500 (http://www.charts.noaa.gov/OnLineViewer/16500.shtml).

Three distinct intertidal areas were sampled: the SAO spill area, a reference area, and a human-impacted area. The latter area, Chernofski Harbor, was a World War II seaplane base outside the spill area (Fig 1). The oiled area included Skan, Makushin, and Portage Bays (Fig 1). The reference area, Pumicestone Bay, was sheltered from the oil slick (Fig 1). Beach data were subdivided into segments at the time of the spill (2004) for assessment, monitoring, and cleanup activity. Three years later, several segments were selected to study lingering oil: of the 23 oiled beach segments under study, 15 (65%) failed to reach final cleanup criteria in 2005 and an additional 3 were subjected to alternative treatment techniques such as berm relocation and tilling. Three types of samples were collected: indigenous mussels, intertidal sediment, and passive samplers deployed in water for about one month. Mussel and passive sampler data were quantitative. Estimation of oil concentration per mass sediment was precluded by patchy oil distribution both within beaches and on individual oiled clasts, hence targeted oil sampling provided only hydrocarbon composition and source information. This study was conducted in a National Wildlife Refuge and permitted by the US Fish and Wildlife Service; it did not involve endangered or protected species.

Beach sampling replicated the methods and coverage of standard shoreline cleanup and assessment techniques completed between 2004 and 2006 [20]. Presence and distribution of surface oil along the entire length of each segment were evaluated by visual survey. Presence and distribution of subsurface oil were evaluated within each identified oiled zone by randomly excavating pits using methods modified from *Exxon Valdez* oil spill studies [21]. Subsurface oil investigation emphasis was on previously identified oiled zones and assumed that previously delineated boundaries represented the area of greatest potential for remaining oil. Pits were allocated to oiled zones proportionally to the zone surface area; the number of pits ranged from 5 (< 100 m^2^) to 80 (≥ 2000 m^2^; S1 Table).

### Sediments

Samples of surface and subsurface oiled sediments were collected from test pits to represent the range of visual oiling conditions and to evaluate the weathering state of remaining oil within each identified oiled zone. Subsurface collection depth ranged from 0 to 110 cm; 89% of the samples were within the upper 50 cm. Because of the coarseness of the sediments (mostly pebbles to boulders), sediment samples consisted of individual clasts with visible oil patches or coating. Where the oil occurred as a specific layer, a composite sediment sample was collected from the oiled layer. Most of the time, the oil occurrence was very patchy; therefore, individual oiled pebbles (4–64 mm) were collected. Volcanic ash, deposited throughout oiled and reference areas by an erupting volcano during the study, was also collected for hydrocarbon analysis for quality assurance purposes. Most of the sediment samples were placed in a pre-cleaned 8-ounce glass jar; large samples (small cobbles greater than 64 mm) were wrapped in aluminum foil and placed with labels in Ziploc bags. The sediments were frozen as soon as practical pending processing.

### Historical Sediment and Oil Samples

Oil (n = 23) and sediment (n = 22) samples collected from beaches oiled by the *S*. *Ayu* about the time of the spill were analyzed to provide an interpretive frame of reference. Frozen samples from the specific beach segments examined in this study were shipped from the original collection agencies (National Oceanic and Atmospheric Administration, Alaska Department of Environmental Conservation, and United States Coast Guard) to the Auke Bay Laboratories. Nearby beach segments were selected to represent oiling for beach segments sampled in 2008 with no previous collections. The median sample date was 27 January 2005 (range 15 December 2004 to 16 May 2005).

### Mussels

Blue mussel (*Mytilus trossulus*) tissue samples were collected using an opportunistic design, with a minimum of three composite invertebrate samples from within, or seaward of each oiled zone, when available. Mussel tissues were collected as a composite of 16 to 25 live mussels 3 to 5 cm in length from an average of three intertidal sites per zone, as close as possible to the shoreline passive sampler arrays, if there was suitable habitat. All tissue sampling was completed prior to tidal inundation of intertidal sediments disturbed during excavation. Live mussels were pried from the substrate by hand wearing clean disposable gloves for each sampling. Whole organisms were wrapped together in aluminum foil and placed in a Ziploc bag with a label on waterproof paper. Upon return to the vessel each day, the sample and label were placed in a second Ziploc bag. The mussel samples were frozen onboard and later shipped overnight to the Auke Bay Laboratories where they remained frozen at −20°C until analysis.

### Passive Samplers (PEMDs)

The bioavailability and composition of mobile oil constituents from remaining oil was assessed in ambient water with low-density polyethylene membrane sampling devices (PEMDs) [14]. These passive samplers were polyethylene plastic strips (~98 μm × 4.9 cm × 50 cm) housed in aluminum canisters (11.5 cm diameter × 6.6 cm) with perforated aluminum endplates (3 mm holes spaced 4.8 mm apart). The PEMDs were placed on ten oiled beach segments within the core spill area and fourteen reference beach segments. The PEMDs were placed in arrays of three, one intertidal and two nearshore. Passive sampling arrays were allocated to oiled zones within beach segments and were proportional to the alongshore length of that zone, except no more than ten arrays were located in a given segment.

The PEMDs were retrieved about one month after deployment (mean 28 days, range 23 to 30 days; 98% were collected within ±2 days of the mean deployment time), sealed in Ziploc bags, and frozen as soon as practical pending processing. Passive sampler air blanks, packed in jars, were opened at each beach segment for about 1 minute either during deployment or retrieval. Two unopened PEMDs served as trip blanks and additional laboratory blanks were never shipped. Shortly after arrival at the laboratory, the aluminum canisters were opened and the PEMDs were transferred to hydrocarbon-free glass jars with Teflon lined lids and frozen for chemical analysis.

### Sample Processing and Hydrocarbon Extraction

Sediment, oil, and mussel samples were spiked with 500 μL of deuterated surrogate recovery standard (S2 Table) and extracted with dichloromethane in a Dionex accelerated solvent extractor [22]. Thawed sediment was homogenized with a spatula before spiking and it was also spiked with 100 μL of a 20 μg/ml solution of a deuterated biomarker recovery surrogate, C27 ααα(20R)-cholestane. Oil samples were homogenized or scraped from rock, if needed, dissolved in dichloromethane, and similarly spiked. Mussel tissue samples were dissected so that the tissue did not contact external shell surfaces and mechanically macerated 3 minutes with a Tekmar tissuemizer before spiking. Extracts were dried with sodium sulfate and concentrated to 1 ml in hexane. Sediment and oil extracts were fractionated into aliphatic and aromatic compounds on chromatography columns (3 g 100% activated silica gel); aliphatic and biomarker compounds were eluted with 6 ml pentane and the aromatic compounds were eluted with 10 ml of a 1:1 mixture of pentane and dichloromethane. Mussel extracts were similarly fractionated (10 g 2% deactivated alumina over 20 g 5% deactivated silica gel); aliphatic compounds were eluted with 50 ml pentane and the aromatic compounds were eluted with 250 ml of a 1:1 mixture of pentane and dichloromethane. Aromatic fractions from tissue were further purified by a high pressure liquid chromatograph equipped with a phenogel size-exclusion column (22.5 mm × 250 mm, 100 angstrom pore size). Aliphatic and aromatic extracts were reduced to 1 ml in hexane, spiked with internal standards, dodecylcyclohexane and hexamethylbenzene, respectively, and stored at −20°C pending analysis.

Passive samplers were wiped clean to remove gross surface contamination, placed in centrifuge tubes, and spiked with 500 μl of a solution equivalent to half the concentration of the deuterated surrogate recovery standard, PAHs only (S2 Table). The spike solvent (hexane) was allowed to evaporate and the PEMDs were extracted in a sonic bath with 100 ml of 80:20 mixture of pentane:dichloromethane for 120 min (three 20 min sonications with a 30 min rest between each sonication). The PEMDs were immediately rinsed with pentane as they were removed after the final sonication. The extracts were dried with sodium sulfate and concentrated to 1 ml hexane. The extracts were purified on a chromatography column (1.5 g 5% deactivated silica gel). Samples were eluted with 22 ml of a 1:1 mixture of pentane and dichloromethane. Extracts were spiked with the internal standard, hexamethylbenzene, and stored at −20°C pending analysis.

All extracts (1 μL) were injected in splitless mode on Aligent model 7890A gas chromatographs (GC) and separated on a 5% phenylmethyl-silicone capillary columns (25 m, 0.20 mm I.D., 0.33 μm film). The GC used for aromatic and biomarker analysis was equipped with a 5975C mass selective detector (MSD). The chromatographic column eluted into the 70 eV electron impact MSD through a 240°C transfer line. The ion source temperature and pressure were 230°C and 10^−6^ torr, respectively.

#### PAH analysis

The aromatic fractions of tissue, sediment, oil, and PEMD extract were injected into the GC at 300°C and the oven temperature ramped from 60 to 300°C at 10°C/min; He carrier gas flow was 1 ml/min. The data were acquired in selected ion monitoring (SIM) mode and concentrations were determined by the internal standard method [14, 23, 24]. Experimentally determined method detection limits were generally 0.7 ng/g for tissue, 0.6 ng/g for sediment, and 0.2 to 3.9 ng/g in PEMDs. The accuracy of the PAH analyses was about ± 15% based on comparison with National Institute of Standards and Technology values, and precision expressed as coefficient of variation was usually less than about 20%, depending on the PAH. Data were analyzed without blank subtraction. Total PAH (TPAH) concentrations were calculated by summing concentrations of individual PAH (S3 Table). Relative PAH concentrations were calculated as the ratio of PAH_*i*_ / TPAH.

Surrogate recoveries varied by analyte and matrix. Recoveries in sediment and passive samplers were fairly uniform, typically about 85 to 86% (95% confidence interval) for all analytes. Naphthalene recovery was less in previously analyzed sediment and tissue (means were 38 and 39%, respectively); means ranged from 52 to 79% for all other analytes. Less than 1% of the recoveries were < 25% (18 of 2754 measurements); all of these were naphthalene and nearly all from tissue (n = 13). All samples were included in the analyses; exclusion of samples with low recovery did not change conclusions.

Hydrocarbon measurements for a few mussel samples were repeated for quality control purposes (n = 8 of 113 samples) and were only used to verify that measurements were repeatable. Evidence of repeatability included high correlation of TPAH concentration and proportions of naphthalenes, fluorenes, dibenzothiophenes, phenanthrenes, chrysenes, and higher molecular weight PAHs; the coefficient of determination (r^2^) ranged from 0.973 to > 0.999.

#### Aliphatic analysis

The aliphatic fractions of tissue, sediment, and oil were injected into the GC at 300°C. The oven temperature remained at 60°C for 1 min, then ramped to 300°C at 6°C/min [23, 24]; He carrier gas flow was 0.8 ml/min. The column eluted into a flame ionization detector. Analyte concentrations were determined by the internal standard method. Experimentally determined method detection limits were generally 19 ng/g for tissue and 10 ng/g for sediment. The accuracy of the alkane analyses was ± 15% based on a spiked blank processed with each set of samples, and precision expressed as coefficient of variation was usually less than about 20%. All surrogate standard recoveries were acceptable (26 to 125%). Total alkane concentrations were calculated by summing concentrations of individual calibrated alkanes (S4 Table). Relative alkane concentrations were calculated as the ratio of alkane_*i*_ / total alkanes.

#### Biomarker analysis

The aliphatic fractions of sediment and oil samples were injected into the GC at 280°C. The oven temperature ramped from 50 to 300°C at 6°C/min; He carrier gas flow was 1 ml/min. The data were acquired in SIM mode, and concentrations were determined by the internal standard method with response factors (RF) based on two representative compounds, 17α(H),21β(H)-hopane (H30) and 5α(H),14α(H),17α(H)-cholestane (C27S). The accuracy of the biomarker analyses was ± 15% based on a spiked blank processed with each set of samples, and precision expressed as coefficient of variation was usually less than about 20%, depending on the biomarker. Biomarker concentrations were not corrected for recovery; surrogate recovery was >70% in 97% of the samples. Total biomarker concentrations were calculated by summing concentrations of individual biomarkers (S5 Table). Relative biomarker concentrations were calculated as the ratio of biomarker_*i*_ / total biomarkers.

### Forensics

The source of hydrocarbons in sediments, mussels, and PEMDs was inferred by multiple methods: 1) oiling records, 2) comparison of biomarker composition in source oil to that in oiled sediment, 3) PAH and alkane composition, and 4) principal components analysis (PCA). Oiling records from the time of the spill through the cleanup process provided primary evidence that any oil discovered in this study likely originated from the *S*. *Ayu*. This evidence included previous standard shoreline cleanup and assessment techniques surveys and associated data. To further inform and authenticate such inferences, oil or oiled sediment samples collected on or nearby specific oiled beaches about the time of the spill were analyzed for comparison to account for possible differences among beaches in source oils or source oil ratios from various *S*. *Ayu* tanks.

#### Hopane, sterane, and triterpane models

Composition of hopanes, steranes, and triterpanes were independently modeled to determine whether composition in sediment samples matched source oil composition. Each sample was scored for fit; if normalized concentrations were between the normalized concentration bounds defined by IFO_L_ and IFO_H_ (or between ±0.05 of those bounds), then that compound was considered a match. This was repeated for each of the 22 hopane compounds. Scores were divided by the number of compounds to yield a proportion. Hopane scores ≥ 0.85 were considered a match to SAO; smaller scores were not a match. Similar comparisons were made among 14 normalized steranes and among 11 normalized triterpanes; the acceptance criterion for each of these was 0.50. Discrimination among sources was best for hopanes; scores for IFO source oil were 1.00 (i.e., 100% fit of 22 compounds). Using ±0.05 relaxed bounds, marine diesel oil from the *S*. *Ayu* scores were 0.16 to 0.21 (n = 2) and anchor tar was 0.32, thus neither of these matched *S*. *Ayu* IFO characteristics. (Anchor tar represented a non-*S*. *Ayu* source applied by a manufacturer to anchors for anti-corrosion purposes. A few of these were deployed above the high-tide line during 2008 sampling.) Discrimination between IFO and anchor tar was poor with steranes and discrimination between IFO and marine diesel oil was poor with triterpanes. Hence, hopane results discriminated best among sources and were used to determine if field samples represented IFO from the *S*. *Ayu*. Hopane modeling results were compared to the originally documented extent and pattern of oil on beaches coated by SAO, visible presence or absence of oil, PAH concentration and PAH model results (see next paragraph), and alkane concentration and composition.

#### PAH model

Hydrocarbon sources in sediment, tissue, and passive samplers were inferred by modeling PAH data. A nonparametric algorithm that recognizes petrogenic and pyrogenic PAH composition was used to ensure interpretation was not confounded by pyrogenic sources; this model is a revision of a previously published model [25]. The PAH model relies on pattern recognition; parent homologues in petrogenic sources are less abundant than alkylated counterparts and concentrations frequently form a rounded ‘hump,’ lower or lowest for the parent compound and peaking somewhere in the alkylated compounds within each homologous group. In contrast, abundance of parent compounds in pyrogenic sources is greatest and concentrations decline with increasing alkylation. Weathering, which is differential molecular size-dependent compound loss, influences these patterns, yet they generally remain discernable.

The PAH model was written as a single unit and is designed to handle petrogenic and pyrogenic results symmetrically. It combines assessment of six homologous families, naphthalenes (N0-N4), fluorenes (F0-F4), dibenzothiophenes (D0-D4), phenanthrenes (P0-P4), fluoranthene-pyrenes (FL, PY, FP1-FP4), and chrysenes (C0-C4). Subscores within any given homologous family range from -1 (pyrogenic) to +1 (petrogenic). The midpoint (0) indicates there was no discernible source. The raw output is summarized as the sum of homologue subscores and the final score is scaled to range from -1 to +1 by dividing by the number of homologous families contributing to the score. See S1 Model for more detail.

#### Principal component analysis (PCA)

Similarities and dissimilarities in hydrocarbon composition were also inferred using PCA. In mussels, PAH concentrations normalized to TPAH concentration were analyzed as a correlation matrix to determine the first two principal components (PCA 1 and PCA 2); C_4_-fluoranthene, C_2_-, C_3_-, and C_4_-chrysenes were not included because they were not detectable. Procedures were the same for PEMDs and biomarkers.

#### Relative quantities of IFOL and IFOH

Hopane composition was used to estimate how much IFO_L_ (hence, also IFO_H_) were in each sample. This was accomplished by examining apparent mixing ratios for each analyte and summarizing the result as a mean. For example, if the normalized concentration in an unknown sample matched the proportion of IFO_L_ in the source oil, then the proportion of IFO_L_ in unknown sample was 1.0. Conversely, if the unknown matched the proportion of IFO_H_, then the proportion of IFO_L_ in the unknown was 0.0. This process was completed on source oils to verify hopane modeling accuracy: the mean estimated IFO_L_ content was 0.82 (range 0.76 to 0.89) in IFO_L_ source oil samples (compared to a theoretical 1.0). Conversely, estimated IFO_L_ content was 0.04 to 0.11 in two replicate IFO_H_ source oil samples (compared to a theoretical 0.0). Thus, assignment of mixing ratios between these two sources is imperfect but reasonable. Dibenzothiophene content relative to phenanthrene content was greater in IFO_H_ than IFO_L_ (S6 Table), thus providing another test of the hopane mixing method. Regression of ΣD/ΣP and estimated IFO_L_ content was significant (P < 0.001, F_o_/F_c_ = 20, r = -0.67, n = 100); that is, proportionate dibenzothiophene content decreased significantly as IFO_L_ content increased.

### Weathering

Data were inspected for evidence of weathering patterns. The PAHs were examined for evidence of weathering by examining the proportion of chrysenes; time-dependent increases in proportional chrysene content were interpreted as evidence of differential loss of smaller aromatic compounds, hence weathering. A weathering index, *w*, was also calculated with a first-order loss rate model [5]. Short and Heintz [5] demonstrated that PAH loss follows first order kinetics;

$$-\frac{d\left[P\right]}{dt}=kf\left(t\right)\left[P\right]$$

where P is a PAH, and the loss rate constant, k, is a function of time (t). Integrating,

$$ln\left(\frac{{\left[p\right]}_{0}}{\left[p\right]}\right)=k\int_{0}^{t}f(t)dt=kw$$

where the value of the integral is indicated by weathering parameter w, which summarizes the exposure history; S1 and S2 Figs illustrate the relationship between PAH composition and *w*.

Time-dependent increases in the largest alkanes (C27 to C36) content (relative to total alkane content) were interpreted as weathering; that is, preferential retention of larger alkanes over time. Biomarker composition was also examined for change; lack of time-dependent change was interpreted as no discernable weathering.

### Statistics

Because some TPAH concentrations were much higher than others, natural log concentrations were compared with ANOVA and geometric mean concentrations are presented for mussel and passive sampler data. Logarithmic transformation helped normalize the data.

## Results

### Hydrocarbon sources and weathering in lingering oil

**Fig 2:**
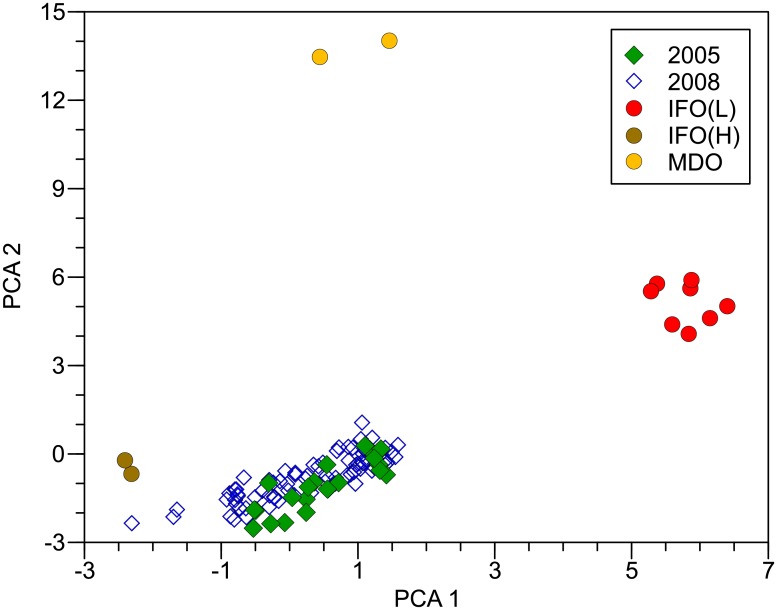
Summary of persistent biomarkers (hopanes and steranes) in oil and sediment. First and second principal components (PCA 1 and PCA 2) from the correlation matrix of normalized persistent biomarkers in all oil and sediment samples collected in 2004 or 2005 (blue) and 2008 (green). Source oils were light and heavy intermediate fuel oil [IFO(L) and IFO(H), respectively]. Marine diesel oil (MDO) was also spilled by the *S*. *Ayu*. References are off-scale low (near PCA 1 = −21). The PCA 1 explained 51% of the variance and PCA 2 explained 19% of the variance.

All previously oiled beach segments contained uniquely identifiable SAO except one (Portage Bay). SAO was positively identified in 2004 and 2005 by hopane, sterane, and triterpane distributions in 21, 21, and 19 of 21 samples, respectively. Results were about the same in 2008, with *S*. *Ayu* IFO mixtures present in 77, 76, or 75 of 78 oiled samples. Hopane and sterane composition in oiled samples in 2004 or 2005 was about the same as in 2008 with no time-dependent separation (Fig 2). Thus, nearly all samples from the oiled area contained SAO, representing all except one beach.

The two primary *S*. *Ayu* source oils, IFO_L_ and IFO_H_, were typically present in nearly equal quantities in oiled sediment samples. Based on hopane distributions in beach samples with likely and confirmed SAO, mean IFO_L_ content was 0.48 (range 0.33 to 0.59; n = 100). Mixture ratios did not vary significantly between years (P_ANOVA_ = 0.863). Because there was no time-dependent biomarker composition change (r^2^ = 0.880; P < 0.001), gradients determined by PCA likely represent a sorting of samples by IFO mixture (Fig 2).

**Fig 3:**
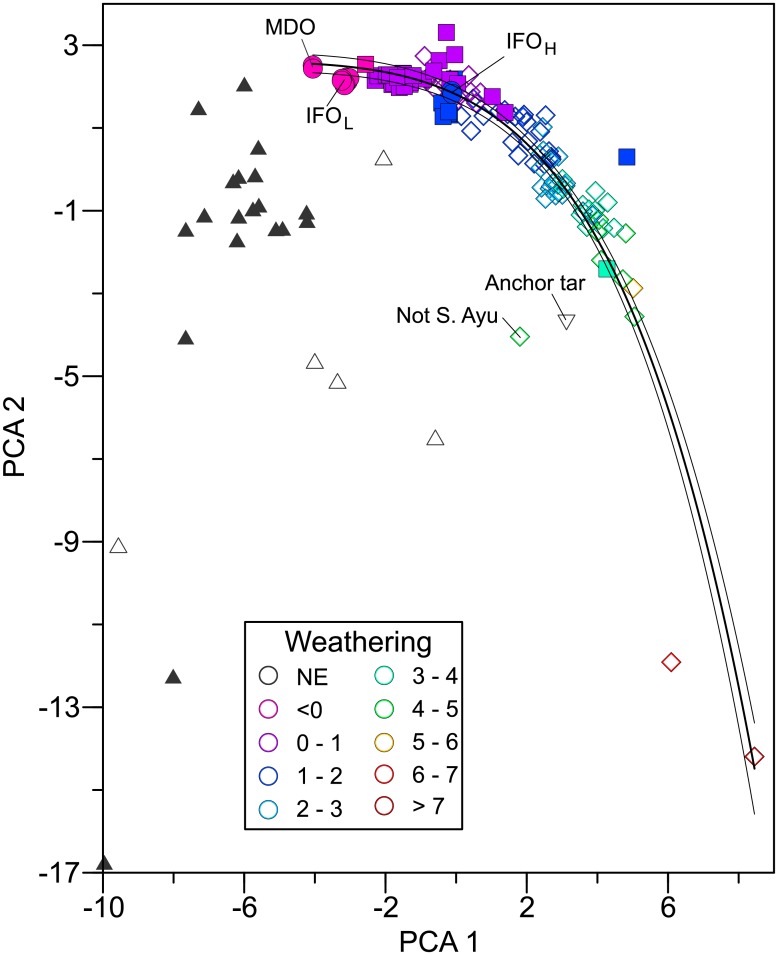
Discrimination of PAHs in reference and oiled samples by principal component analysis. *S*. *Ayu* source oil is illustrated with solid circles. Source oil types were marine diesel oil (MDO), intermediate fuel oil light (IFO_L_), and intermediate fuel oil heavy (IFO_H_). Solid squares indicate oil from sediment in 2004–2005 and open diamonds indicate oil from 2008 sediment. Black triangles indicate non-oiled reference samples and open triangles represent 2008 data; weathering was not estimated (NE) in these. Anchor tar from an unknown origin is also included for comparison. One sample with hydrocarbons from the oiled area was not verified as *S*. *Ayu* oil (annotated). Percent total chrysenes in oiled samples is indicated by color; small values indicate the least weathered samples, large values indicate the most weathering. The first component (PCA 1) explained 28% of the variance and the second (PCA 2) explained 21% of the variance.

**Fig 4:**
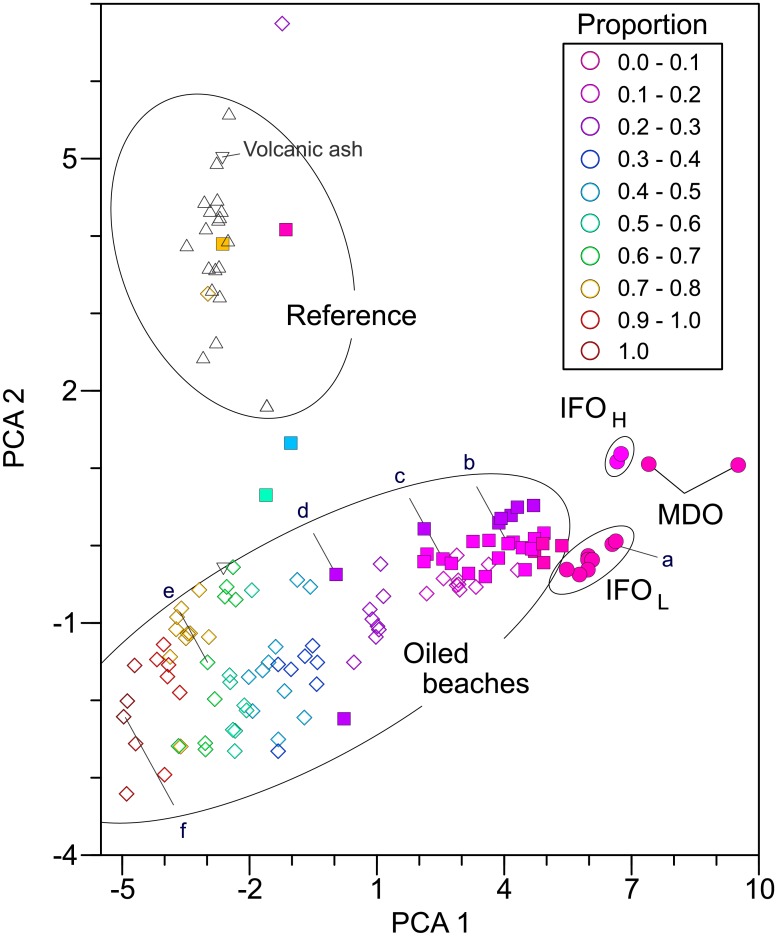
Discrimination of alkanes in reference and oiled samples by principal component analysis. Oiled samples are color coded by the proportion of heavy alkanes (≥*n*-C27 alkanes). *S*. *Ayu* source oil is illustrated with solid circles. Source oil types were marine diesel oil (MDO), intermediate fuel oil light (IFO_L_), and intermediate fuel oil heavy (IFO_H_). Solid squares indicate oil from sediment in 2004–2005 and open diamonds indicate oil from 2008 sediment. Black triangles indicate non-oiled reference samples; open triangles represent 2008 data. Letters (a–f) indicate samples illustrated as examples in S3 Fig. The PCA 1 explained 40% of the variance and PCA 2 explained 14% of the variance.

The presence of lingering SAO in Unalaska beaches was further substantiated by PAH and alkane composition. Every sample identified with SAO using biomarkers was identified by PAH modeling as petrogenic (n = 99; including samples from 2004 through 2008). These same samples all had alkane distributions and unresolved complex mixtures consistent with oil. All samples with visible oil contained SAO; three additional trace oil samples collected in 2005 from oiled areas had PAH composition consistent with oil but no confirmatory biomarker analyses were completed. PAH modeling provided no evidence of oil in reference samples (n = 19). All reference samples were distinguished from oiled samples by PCA (Figs 3 and 4).

The PAHs in lingering SAO weathered, indicated by time-dependent change in composition. This change was detected by *w* [5], change in content of the most persistent PAHs, and with PCA (Fig 3). Weathering increased significantly from 2004 and 2005 (*w* = 0.66) to 2008 (*w* = 2.59; P_ANOVA_ < 0.001). The proportion of total chrysenes increased significantly from 2004 and 2005 (0.03) to 2008 (0.07; P_ANOVA_ < 0.001), consistent with proportionately better retention of this environmentally persistent group than smaller PAHs. The proportion of total chrysenes was correlated with *w* (r = 0.938, linear regression, n = 119) and with PCA 1 (r = 0.942, exponential regression, n = 123; Fig 3). Thus, the patterns evident in PCA are related to weathering (more rapid loss of smaller PAHs; Fig 3).

Alkanes in lingering SAO weathered, indicated by time-dependent change in composition. This change was detected by change in content of the most persistent alkanes and with PCA (Fig 4). Alkanes were less weathered in 2004 and 2005 samples than in 2008 samples; the proportion of environmentally persistent alkanes (*n*-C27 and above) increased significantly from 0.69 to 0.96 (P_ANOVA_ < 0.001). Furthermore, the proportion of environmentally persistent alkanes was least in source oil and greatest in samples least similar to source oil (Fig 4). Moreover, the proportion of environmentally persistent alkanes was correlated with PAH weathering (r = 0.798, linear regression, n = 106), percent chrysenes (r = 0.905, reciprocal exponential regression, n = 112), and PCA 1 (r = -0.959, cubic regression, n = 112). Alkane composition in reference samples was distinctly different from that in oiled samples (Fig 4).

### Background hydrocarbons in sediment

Hydrocarbon sources in background sediment were not apparent. Total PAH concentrations were low (typically < 1 ng/g dry wt in upstream river sediment and 1.4 ng/g in volcanic ash). Composition was ambiguous; PAH model results ranged from -0.1 to 0.2 with no evidence of either pyrogenic or petrogenic sources (n = 19). Napthalenes were the most common PAHs. Perylene was present in 8 samples, likely contributed by contemporary sediment diagenesis [26]. Odd-chain alkanes dominated, indicative of plant sources [27, 28]. Conditions were similar in reference marine sediment except perylene was not detected (TPAH < 0.5 ng/g, PAH model range 0 to 0.1, n = 3). Triterpanes, hopanes, and steranes were not detected in contemporary volcanic ash.

### Bioavailability

#### PAHs in mussels

**Fig 5:**
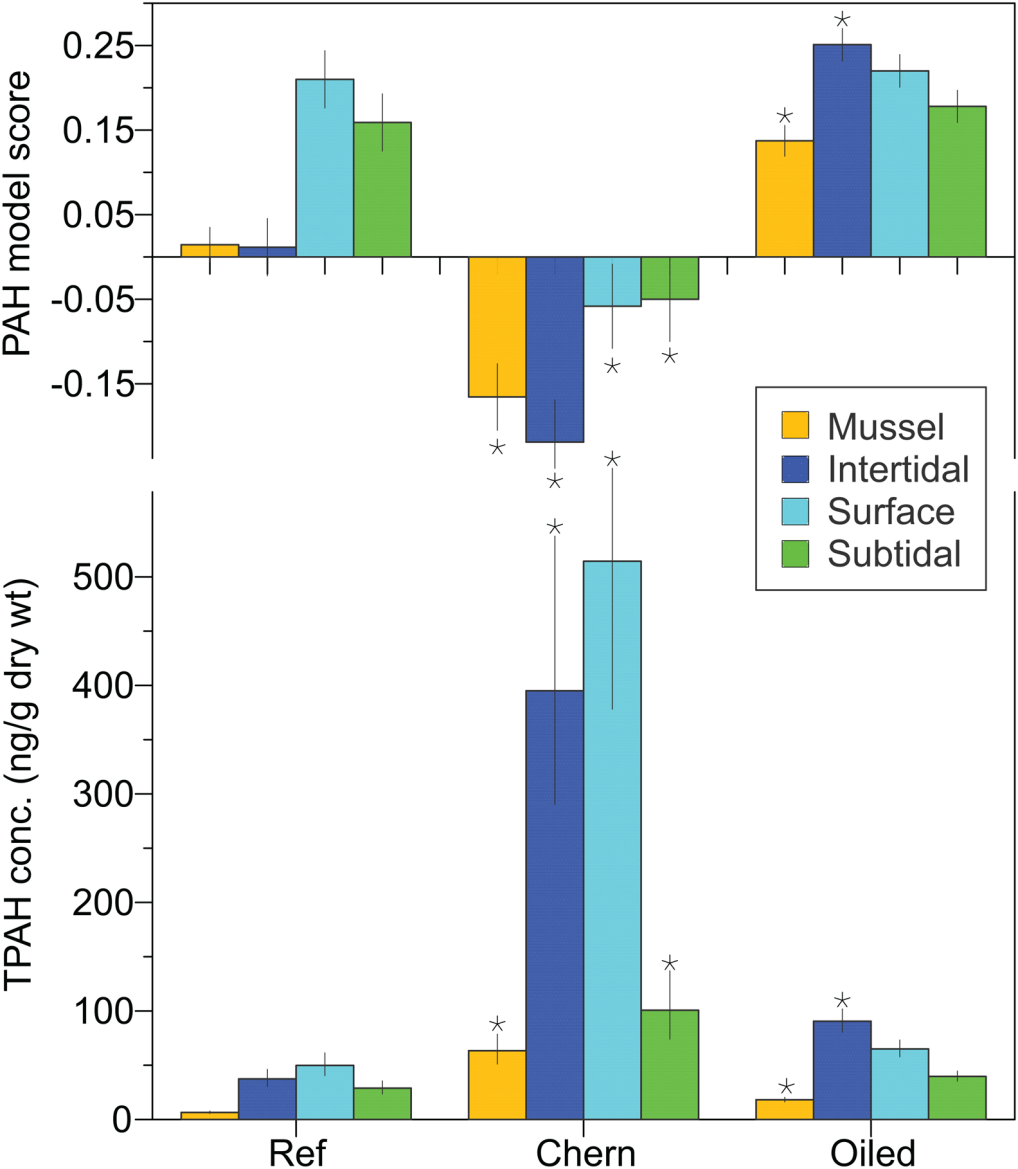
Mean TPAH and PAH model scores in mussels and PEMDs by area [reference (ref), Chernofski Harbor (Chern), and oiled] and zone (subtidal, surface water, and intertidal). Potential PAH model scores range from -1 (pyrogenic) to +1 (petrogenic); scores close to 0 indicate ambiguous sources. Error bars are ±SE. Statistically significant results (versus reference values) are indicated with asterisks. Samples were collected in 2008.

Composition of PAHs in mussel tissue collected in 2008 differed between oiled, reference, and human-impacted areas, detectable with PAH modeling and with PCA (biomarkers were not measured). Composition was significantly more petrogenic in the oil spill area; pyrogenic sources were most likely in Chernofski Harbor (P_ANOVA_ < 0.001; Fig 5).

**Fig 6:**
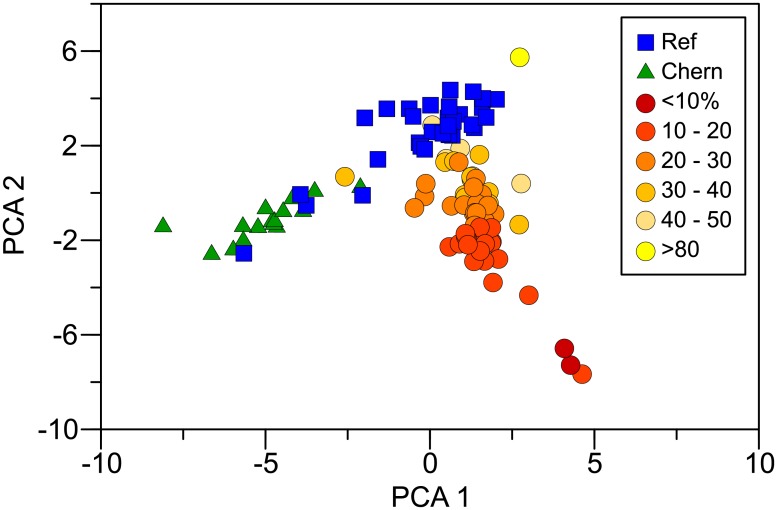
First and second principal components (PCA 1 and PCA 2) from the correlation matrix of normalized PAHs in mussel tissue. Oiled sites are illustrated with circles, reference with squares (blue), and Chernofski Harbor with triangles (green). Oiled results are color coded by percent naphthalenes, a measure of weathering. The most weathered samples have the least naphthalene.

Consistent with the differences among areas detected by PAH modeling, PCA separated PAH composition in mussels into three distinct groups with limited overlap: Chernofski Harbor, the reference area, and the oiled area (Fig 6). Although PCA 1 and PCA 2 only explained 31% of the variance, they corroborated the composition differences determined with PAH modeling. Change in homologue proportions provided an estimate of weathering in mussel tissue (more naphthalenes indicate less weathering). Naphthalenes proportions were inversely correlated with the second PCA component (r = -0.879, two-phase exponential decay regression) and chrysenes proportions were positively correlated with it (r = 0.549, linear regression; P < 0.001). Thus, differences in PAH composition in mussels from oiled and reference areas can be explained by the presence of variably weathered oil in the former (Fig 6).

Total PAH concentration also distinguished mussels from reference, oiled, and previously contaminated areas. The TPAH concentrations in mussels in the historical human impact area (63 ng/g dry weight, Chernofski Harbor) were significantly greater than in reference areas (6.5 ng/g; P_ANOVA_ < 0.05; Fig 5). Mean TPAH concentrations in the oil spill area (18 ng/g) were also greater than in the reference area (P < 0.05; Fig 5).

#### PAHs in passive samplers (PEMDs)

Composition of PAHs in PEMDs deployed in 2008 differed between oiled and reference areas, detectable with PAH modeling and with PCA. Petrogenic sources were significantly more likely intertidally in the spill area than in the reference area (P_ANOVA_ < 0.05; Fig 5). A pyrogenic source was significant in Chernofski Harbor (P_ANOVA_ < 0.05; Fig 5). The spatial distribution of PAH sources in PEMDs was similar to that in mussels and PAH sources were correlated between mussel and PEMD pairs (r = 0.562; P < 0.001; linear regression).

**Fig 7:**
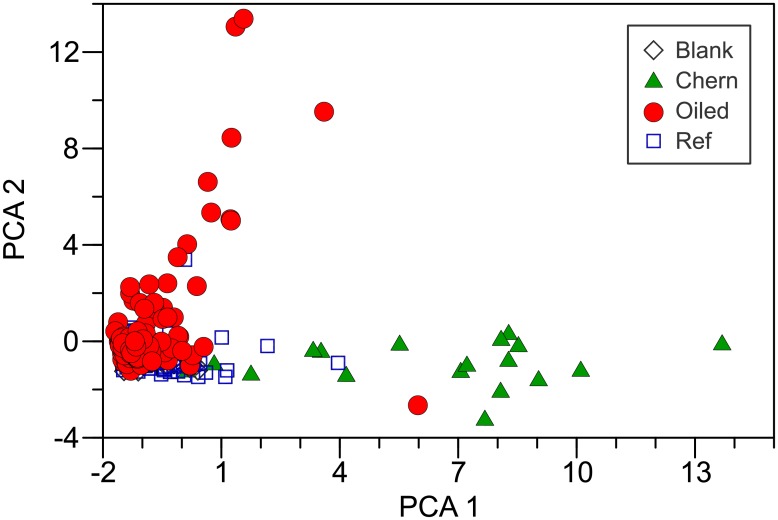
First and second principal components (PCA 1 and PCA 2) from the correlation matrix of normalized PAHs in passive samplers (PEMDs).

PCA analysis confirmed that PAH composition differed among areas. All PCA scores from all areas overlapped those in blanks (Fig 7). References were generally closely adjacent to blanks. Composition in the oiled group extended well beyond the reference group with positive change in the PCA 1 and PCA 2 directions. Composition in the Chernofski Harbor group extended positively only in the PCA 1 direction. Thus, there were distinct differences among groups as well as commonalities. These patterns were also evident when the analysis was subdivided by position (intertidal, subtidal, and surface water).

Total PAH concentration also distinguished PEMDs among areas. Mean concentrations were consistently highest in Chernofski Harbor and lowest in reference areas. In the oiled area, TPAH concentration was significantly elevated intertidally (P < 0.05) compared to the reference concentration (Fig 5). In Chernofski Harbor, TPAH concentrations were significantly elevated in all three zones (P < 0.05; Fig 5). Concentration differences among areas were least for the subtidal samples (Fig 5).

Relative TPAH concentration patterns in passive samplers were the same as those in mussels (Fig 5). In addition, TPAH concentrations in intertidal PEMDs were related to those in paired mussel samples (P_linear regression_ < 0.001; n = 53), though two high concentration pairs in Chernofski Harbor were leverage points. Without these, P = 0.052 and correlation was weak (r = 0.273).

## Discussion

Biologically available oil was discovered in the *S*. *Ayu* spill area 3.6 y after the spill. This oil contained toxic tricyclic aromatic constituents [29], demonstrated by accumulation of PAH in mussels and passive samplers from oiled areas. These accumulations were distinguished from reference samples by composition and concentration. The PAH modeling indicated a petrogenic source in the oiled area and distinct differences in PAH composition among areas was confirmed with PCA. In passive samplers, PAH concentrations in the oiled area were significantly elevated intertidally and in surface water and were correlated with those in nearby mussels. A petrogenic source was consistently and significantly more likely in passive samplers deployed in the oiled area than elsewhere. The sources of these biologically available PAHs were likely the hydrocarbons present in corresponding shorelines.

Multiple lines of evidence converged to identify *SAO* in all oiled beach segments examined in 2008 except Portage Bay. Hopane composition provided the most definitive proof that *SAO* was present; composition matched *S*. *Ayu* IFO composition in 77 of 78 oiled samples. Composition and concentration of PAHs and alkanes, concentration of the unresolved complex mixture, plus sample locations and appearances all supported the conclusion that *SAO* was present in these 78 field samples. There was no evidence that potentially introduced hydrocarbons from tarred anchors or support vessels contributed to the hydrocarbons observed in environmental samples. In addition, observation of biologically available oil is corroborated by other studies, including evidence that exposure continued in harlequin ducks and mussels for 3 y [3, 4].

Amounts of bioavailable oil declined since the *S*. *Ayu* spill, consistent with other spill experiences. Total PAH concentrations in mussels declined exponentially from 2005 to 2008 [3, 4]. PAH modeling (by our group) consistently provided the strongest indications of oiling nearest the time of the spill. Likewise, the mean TPAH concentration in passive samplers deployed in oiled bays in 2005 was about 10,000 ng/device [30], roughly 100 times greater than concentrations in 2008. Declines in TPAH concentration in mussel tissue were also exponential after the *Exxon Valdez* oil spill [31, 32], consistent with these observations.

Weathering of *SAO* was the most likely source of biologically available hydrocarbons at previously oiled beaches. Thermodynamically driven loss of hydrocarbons from oil to water and from water to organism (or passive sampler) is likely a primary contamination mechanism. Differential loss of smaller molecules is defined as weathering and that process explains the observed time-dependent changes in PAH and alkane composition. The PAH composition changes between 2005 and 2008 were consistent with weathering; for example, the proportion of chrysenes, which are relatively large and environmentally persistent increased significantly. By 2008, most samples were more weathered than could be explained by composition of the most weathered source oil (IFO_H_), thus the statistically significant time-dependent change was caused by active weathering. Similarly, time-dependent shifts in alkane composition were significant, consistent with weathering, and exceeded weathering in all source oils, therefore could not be explained as an unchanging consequence of source oil mixing.

Principal component analysis allowed examination of weathering processes in PAHs and alkanes and provided an unbiased method to distinguish oil sources and non-oiled samples. The well-documented weathering of PAHs [8, 10, 13, 33], summarized by *w* [5], was related to principal components, demonstrating the mathematical link between the two approaches. Increased PAH weathering can also be described by increased proportions of the most persistent PAHs such as chrysenes—and weathering and principal components were related to chrysene proportions. Furthermore, the results directly support a relationship between PAH and alkane weathering; preferential loss of smaller alkanes was obvious (S3 Fig), consistent with known weathering patterns [34, 35]. In addition, weathering of smaller biomarker molecules (isoprenoids, which are also classified as alkanes) was also related to PAH weathering and loss patterns were consistent with known biomarker weathering [34]. Distribution patterns in principal component space were essentially the same for each class of compounds; the least weathered oils, collected directly from the *S*. *Ayu*, were at one extreme and each point cloud extended away, ending with the most weathered patterns in each compound class. Clear compositional gradients existed across each distribution, hallmarks of weathering and in every case PCA 1 was correlated with weathering metrics. The significant time-dependent shifts along these weathering gradients confirmed that the PCA analysis detected active weathering and was not simply sorting data based on unchanging mixtures of source oil. Thus, PCA provided a consistent framework to understand and explore these data and, coupled with changing composition patterns, demonstrated PAH and alkane weathering.

Unlike the time-dependent weathering in PAHs and alkanes, biomarkers (triterpanes, hopanes, and steranes) did not weather during the 3 y observation period. Biomarker composition in 2008 was essentially identical to composition in 2005 with no evidence of change. Composition of these compounds varied among source oils, notably between the two largest reservoirs (IFO_L_ and IFO_H_), and composition in environmental samples reflected mixtures of these two sources, generally about half of each. In this case, the gradients evident in PCA reflected unchanging biomarker mixtures and composition did not shift between 2005 and 2008 (Fig 2). Biomarker composition in spilled marine diesel oil was clearly different than in IFO but when relative oil volumes (96% IFO, 4% marine diesel oil), densities (0.964 and 0.852 g/ml for IFO and marine diesel oil, respectively), and total biomarker content in each source are accounted for, the potential influence of marine diesel oil on composition in spilled oil was < 1% and was not detected.

The unchanging biomarker composition provided a definitive tool for source identification. Of the three biomarker classes, hopane composition provided the most definitive and consistent information; emphasis on these compounds in an alternative analytical approach (Nordtest [36]) is appropriate. However, we caution that the purpose of the Nordtest is to identify oil sources and that the ratios selected are designed to be unchanging, thus the Nordtest approach cannot be used to understand weathering processes. Examination of hopanes discriminated among all oil sources encountered in thus study; IFO, MDO, anchor tar, and background. It also discriminates IFO from another source oil spilled in Alaskan waters, Alaska North Slope crude oil.

Toxic PAH constituents were present in oil in 2008 and these molecules were mobilized from SAO by weathering. For example, oil in sediments contained fluorenes, dibenzothiophenes, phenanthrenes, and pyrenes, all toxic to fish embryos [37]. Weathering removes lower molecular weight compounds most rapidly, preferentially leaving the heavier and more toxic PAHs (compare S1 and S2 Figs), yet weathering was not so extensive that mobile, intermediate-sized, toxic PAHs were gone from the oil. Furthermore, measured PAH concentrations in the oil were above previously established method detection limits in most samples (93%), thus verifying the presence of oil.

The mobility, hence bioavailability, of toxic constituents from SAO is supported by mussel and passive sampler data, confirming the differential loss of oil constituents as dictated by thermodynamics [5]. Toxic PAHs were evident in mussel tissue, though generally at low concentrations. PAH modeling did not unambiguously identify oil in mussels from oiled areas, rather only indicated a tendency towards oil compared to reference mussels. However, aqueous transfer and subsequent uptake by living organisms tends to modify PAH composition, making interpretation more difficult [13, 25]. Less hydrophobic compounds leave oil and enter water more rapidly than more hydrophobic compounds, explaining weathering and resulting in an aqueous PAH composition biased toward lower molecular weight PAHs with respect to oil [5, 14]. Conversely, living organisms (and passive samplers) preferentially accumulate the more hydrophobic PAHs and animals, including mussels, are capable of metabolizing PAHs [38]. Composition of PAHs in oiled mussels suggested the presence of oil constituents; in particular, phenanthrene distributions in tissue were frequently consistent with a petroleum source. Oil patterns were also apparent in the less frequently observed fluorenes, dibenzothiophenes, and fluoranthene-pyrenes.

Consistent corroboration among data sets demonstrates that meaningful chemical information is present at low PAH concentrations. This correspondence included corroboration of mussel data by passive samplers and vice versa including similarity of TPAH concentrations, PAH model estimates, and area separation by principal components analysis. Furthermore, the low-level patterns in passive samplers were repeated in three different zones, intertidal, subtidal, and surface water. In addition, a consistent relationship between principal components and weathering emerged in mussels and passive samplers that was consistent with the same pattern in whole oil samples, where concentrations typically substantially exceeded method detection limits. Further corroborative evidence was provided by the existence of a third unique signature in a historically contaminated area (Chernofski Harbor). Moreover, the same pattern of petrogenic sources in oiled areas and a tendency toward pyrogenic sources in Chernofski Harbor was evident in mussel samples collected the previous winter and independently analyzed by a different laboratory (Alpha Woods Hole) [4].

A third distinct, historically impacted area was discovered during the chemical survey, separated from the reference area by greater mean PAH concentrations and pyrogenic composition. Chernofski Harbor was likely contaminated as a result of World War II activities when it was used as a seaplane base; the area was also used for sheep ranching. As of 2005, the military cleanup status of Chernofski Harbor was pending [39]. Remnants of buildings and docks are evident in the area. Perhaps creosote used to preserve pilings may explain at least in part the pyrogenic signature observed in this harbor, an inference strengthened by a mussel sample collected directly from a wooden piling with a clear pyrogenic pattern and order of magnitude higher TPAH concentration than in any other sample.

The multi-year persistence of intertidal oil deposits from the *S*. *Ayu* and continued bioavailability of PAHs are consistent with other spill histories [32, 40–52]. Oil effects can persist for many years in sediment [32, 40–42]. For example, intertidally deposited *Exxon Valdez* oil has persisted for more than 20 y and in some places is nearly as toxic as it was the first few weeks after the spill [42–45]. Oil spilled from the oil barge *Florida* in West Falmouth, Massachusetts, has persisted > 30 y in marsh sediment and will likely persist indefinitely; toxic, substituted triaromatic and higher ring number aromatics are degraded slowly [46, 47]. Hydrocarbons from the *Florida* induced cytochrome P4501A in mummichogs (*Fundulus heteroclitus*) 8–20 y later, signaling continued biological availability and incomplete habitat recovery [48, 49]. Estimated residence time for VLCC *Metula* oil in the Strait of Magellan was 15–30 y in low energy sand and gravel beaches and >100 y in sheltered tidal flats and marshes [50]. Weathered Bunker C oil was detected in Chedabucto Bay, Nova Scotia, 20 y after the SS *Arrow* spill [51]. After 6–7 y, clams (*Mya arenaria*) from an oiled location in Chedabucto Bay remained stressed and species diversity was uniformly lower at oiled locations than at reference locations [52].

Bioaccumulation from aqueous sources was evident after the Deepwater Horizon oil spill. For example, Mitra et al observed PAH bioaccumulation in mesozooplankton in the northern Gulf of Mexico in September 2010 after the Macondo well was capped [53]. Sublethal exposures to Macondo oil altered genome expression and physiological impairment in estuarine killifish (*Fundulus grandis*) for more than a year [54–56]. In the context of an ecosystem, where multiple natural stressors present challenges to organism, the additional challenge of PAH bioaccumulation (or other toxins) can be amplified and contribute to impacts on fitness, populations, and communities beyond those predicted from direct hydrocarbon toxicity [57]. Microbial communities can also be altered by aqueous PAH exposure; functional composition and structure were dramatically altered in the deep-sea Deepwater Horizon oil plume [58]. We expect, but cannot prove, that epibenthic organism exposure in habitat contaminated by the *S*. *Ayu* was nearing the lower limit of detectability in 2008 because concentrations in mussels were declining exponentially [4]. We observed the same pattern in Prince William Sound after the Exxon Valdez oil spill; mussel exposure declined to background levels despite continued presence of oil sequestered in intertidal sediment.

In summary, hydrocarbons lost from one compartment (whole oil) were detectable in another (mussels and passive samplers) implying aqueous transfer. Lower molecular weight molecules, including PAHs and alkanes were differentially lost from stranded SAO whereas the largest compounds including triterpanes, hopanes, and steranes were not lost from the oil during the 3.6 y observation period. This weathering process is the most probable mechanism for observed biological exposure (and sequestration in passive samplers). Spillage of nearly equal quantities of two distinct source oils helped determine that observed biomarker mixtures did not vary with time, hence environmentally persistent biomarkers were used to definitively identify stranded oil. Molecules lost from stranded oil were discovered in indigenous mussels and passive samplers deployed 3.6 y after the spill. Although uptake varied from negligible to substantive in the oiled area, concentration and composition were significantly different than in the reference area and patterns observed in mussels were repeated in passive samplers deployed in three zones (intertidal, subtidal, and water).

## Supporting Information

S1 Fig Relative polynuclear aromatic hydrocarbon (PAH) composition in IFO_L_ *S*. *Ayu* oil samples collected between December 19, 2004 and January 5, 2005 from least weathered (a) to most weathered (d). Vertical bars indicate ± 1 standard error (2 ≤ n ≤ 3). See S3 Table for abbreviations. See text for an explanation of the unitless weathering coefficient *w*.
(EPS)

S2 Fig Relative polynuclear aromatic hydrocarbon (PAH) composition in representative sediment samples collected in 2008 from least weathered (a) to most weathered (f). See S3 Table for abbreviations. See text for an explanation of the unitless weathering coefficient *w*.
(EPS)

S3 Fig Relative alkane composition in oil collected from the *S*. *Ayu* and oil or oiled sediment from beaches, 2004 to 2008. See S4 Table for abbreviations. PCA 1 is the first principal component in the analysis illustrated in the primary paper (Fig 4).
(EPS)

S1 Model Nonparametric PAH modeling. (DOCX)

S1 Table Allocation of pits to oiled zone area for the purpose of exploring for buried oil. (DOCX)

S2 Table Deuterated surrogate polynuclear aromatic hydrocarbon (PAH). (DOCX)

S3 Table Polynuclear aromatic hydrocarbon (PAH) analytes, abbreviations, deuterated surrogate references, molecular mass, and the log of the octanol-water partition coefficient (K_ow_). (DOCX)

S4 Table Measured alkanes and their abbreviations. (DOCX)

S5 Table Biomarker abbreviations and target ions (m/z). (DOCX)

S6 Table Attributes of source oils; intermediate fuel oil heavy (IFO_H_), intermediate fuel oil light (IFO_L_), and marine diesel oil (MDO). (DOCX)
