# Supplementary material for: Spilled Oils: Static Mixtures or Dynamic Weathering and Bioavailability?
Source: PLoS One. 2015 Sep 2;10(9):e0134448. doi: 10.1371/journal.pone.0134448 (PMC4557949; doi:10.1371/journal.pone.0134448)

**S1 Model**

Carls (2006) previously demonstrated that nonparametric modeling designed to discriminate petrogenic and pyrogenic sources provides an unbiased assessment of polynuclear aromatic hydrocarbon (PAH) composition in oils, tissue, passive samplers, and other matrices ([1](#_ENREF_1)). Application of these methods aids the data analysis process and model results can be analyzed to discover differences (or not) among geographic areas, experimental treatments, or other study design structures.

The model used for analysis in the associated paper is functionally a revision and extension of a previously published nonparametric source model ([1](#_ENREF_1)). The original model is a combination of two nonparametric algorithms, one to estimate pyrogenic content and one to estimate petrogenic content plus two other independent models ([2](#_ENREF_2), [3](#_ENREF_3)). The first nonparametric algorithm was designed to find petrogenic signals based on the observation that unsubstituted parent homologue concentrations are typically smaller than alkyl-substituted concentrations and the second algorithm was designed to find pyrogenic relationships based on the opposite homologue relationship. The original petrogenic model increased scores for the presence of five homologous families [naphthalenes (N0-N4, where N0 is the parent compound and N1-N4 are alkylated naphthalenes), fluorenes (F0-F3), dibenzothiophenes (D0-D3), phenanthrenes (P0-P4), and chrysenes (C0-C1)] and where *X*_0_*_j_* < *X_ij_ ;* *X_ij_* represents each homologous family ( *j,* range 1-5) , and homolog within family (*i, range* 1-*n_j_)*, where *n_j_* is the number of homologues in the *j*^th^ family (4, 3, 3, 4, and 1, respectively) ([1](#_ENREF_1)). The original pyrogenic model was based on the observation that *X*_0_*_j_ »* *X_ij_* in PAH from pyrogenic sources and assigned weighted scores ([1](#_ENREF_1)). Results of these two original models were added and this result was further combined with two other models to assess composition. In practice the original model functioned well at detecting petrogenic sources but was not as effective at detecting pyrogenic sources, both as a result of inclusion of the external models and the way the nonparametric pyrogenic algorithm was written. Thus, a single nonparametric algorithm was written to replace analogous functions in the original and the new model does not include calculations by other authors.

This nonparametric model was written as a single unit and is designed to handle petrogenic and pyrogenic results symmetrically. It combines assessment of six homologous families, naphthalenes (N0-N4), fluorenes (F0-F4), dibenzothiophenes (D0-D4), phenanthrenes (P0-P4), fluoranthene-pyrenes (FL, PY, FP1-FP4), and chrysenes (C0-C4), and thus considers more data than the original model. Scores within any given homologous family range from -1 (pyrogenic) to +1 (petrogenic). The midpoint (0) indicates there was no discernible source. The raw output is summarized as the sum of homologue scores, thus ranges from -6 (pyrogenic) to +6 (petrogenic). The final score is scaled to range from -1 to +1 by dividing by the number of homologous families contributing to the score. The model also reports individual homologue results and flags samples with mixed results (i.e., those with both pyrogenic and petrogenic characteristics), thus allowing a detailed view of model function and opportunity to focus on promising subsets where results may otherwise be complicated. The functioning model exists as a series of cell formulae in Excel.

The model relies on pattern recognition; parent homologues in petrogenic sources are less abundant than alkylated counterparts and concentrations frequently form a rounded ‘hump,’ lower or lowest for the parent compound and peaking somewhere in the alkylated compounds within each homologous group (Fig. A). In contrast, abundance of parent compounds in pyrogenic sources is greatest and concentrations decline with increasing alkylation (Fig. A). Weathering, which is differential molecular size-dependent compound loss, influences these patterns, yet they generally remain discernable.

The model unambiguously discriminates among pyrogenic and petrogenic samples and yields intermediate results in simulated mixtures. Model estimates were 1.0 and −1.0 for the oil and creosote examples illustrated in Fig. A. Model results were 1.0 ± 0.0 for 21 SRM 1582 analyses and 1.0 ± 0.0 for 21 Alaska North Slope oil samples. Model results were −0.67 in two creosote samples collected by our group; naphthalene patterns were petrogenic in both but composition was pyrogenic in all other homologs. In mathematically simulated mixtures, model results transitioned smoothly from −1.0 (pyrogenic) to +1.0 (petrogenic; Fig. B).

A functioning version of the model is available from the corresponding author upon request.

**Fig. A.** Petrogenic and pyrogenic polynuclear aromatic hydrocarbon composition patterns in oil (NIST SRM 1582, top panel) and creosote (bottom panel).

**Fig. B.** Example changes in model scores with simulated mixtures of two sources. One of these sources was creosote, the other either fresh *Exxon Valdez* crude oil (blue circles and curve) or weathered *Exxon Valdez* crude oil (green squares and curve).


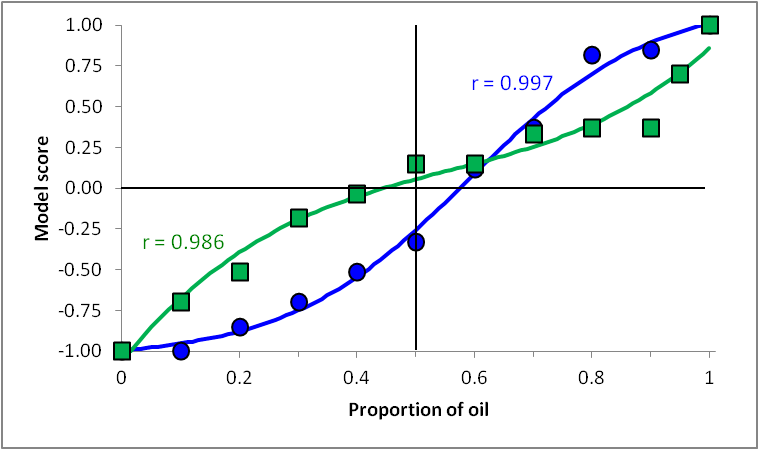

Supplement: S1 Model — (DOCX) [file pone.0134448.s004.docx]
