# Supplementary material for: Spilled Oils: Static Mixtures or Dynamic Weathering and Bioavailability?
Source: PLoS One. 2015 Sep 2;10(9):e0134448. doi: 10.1371/journal.pone.0134448 (PMC4557949; doi:10.1371/journal.pone.0134448)
Supplement: S1 Table — (DOCX) [file pone.0134448.s005.docx]

**S1 Table.**

| Oiled Zone area (m^2^) | Zone Count | N Pits |
| --- | --- | --- |
| 0 to <100 | 1 | 5 |
| 100 to <500 | 11 | 10 |
| 500 to <1,000 | 2 | 20 |
| 1,000 to <2,000 | 10 | 40 |
| ≥2,000 | 3 | 80 |
