# Supplementary material for: Spilled Oils: Static Mixtures or Dynamic Weathering and Bioavailability?
Source: PLoS One. 2015 Sep 2;10(9):e0134448. doi: 10.1371/journal.pone.0134448 (PMC4557949; doi:10.1371/journal.pone.0134448)
Supplement: S2 Table — (DOCX) [file pone.0134448.s006.docx]

**S2 Table.**

| **(μg/ml)** | **Surrogate** |
| --- | --- |
| 2.0 | naphthalene-d_8_ |
| 2.0 | acenaphthene-d_10_ |
| 2.0 | phenanthrene-d_10_ |
| 2.0 | chrysene-d_12_ |
| 2.0 | perylene-d_12_ |
| 2.0 | benzo[*a*]pyrene-d_12_ |
| 9.9 | n-dodecane-d_26_ |
| 9.7 | n-hexadecane-d_34_ |
| 9.7 | n-eicosane-d_42_ |
| 9.8 | n-tetracosane-d_50_ |
| 9.7 | n-triacontane-d_62_ |

Spike volumes were 500 μL for tissue and sediments, and half the equivalent for PEMDs with PAHs only. Spike solvent was hexane.
