# Supplementary material for: Spilled Oils: Static Mixtures or Dynamic Weathering and Bioavailability?
Source: PLoS One. 2015 Sep 2;10(9):e0134448. doi: 10.1371/journal.pone.0134448 (PMC4557949; doi:10.1371/journal.pone.0134448)
Supplement: S3 Table — (DOCX) [file pone.0134448.s007.docx]

**S3 Table**

| **PAH** | **Abbreviation** | **Surrogate** | **Molecular mass (g/mole)** | **log *K*_ow_** |
| --- | --- | --- | --- | --- |
| naphthalene | N0 | 1 | 128.2 | 3.36 |
| C-1 naphthalenes | N1 | 1 | 142.2 | 3.80 |
| C-2 naphthalenes | N2 | 2 | 156.2 | 4.30 |
| C-3 naphthalenes | N3 | 2 | 170.3 | 4.80 |
| C-4 naphthalenes | N4 | 2 | 184.3 | 5.30 |
| biphenyl | BIP | 2 | 154.2 | 3.80 |
| acenaphthylene | ACN | 2 | 152.2 | 3.22 |
| acenaphthene | ACE | 2 | 154.2 | 4.01 |
| fluorene | F0 | 2 | 166.2 | 4.21 |
| C-1 fluorenes | F1 | 2 | 180.3 | 4.72 |
| C-2 fluorenes | F2 | 2 | 194.3 | 5.20 |
| C-3 fluorenes | F3 | 2 | 208.3 | 5.70 |
| C-4 fluorenes | F4 | 2 | 222.3 | *6.20 |
| dibenzothiophene | D0 | 3 | 184.2 | 4.53 |
| C-1 dibenzothiophenes | D1 | 3 | 198.3 | 4.96 |
| C-2 dibenzothiophenes | D2 | 3 | 212.3 | 5.42 |
| C-3 dibenzothiophenes | D3 | 3 | 226.3 | 5.89 |
| C-4 dibenzothiophenes | D4 | 3 | 240.3 | *6.34 |
| phenanthrene | P0 | 3 | 178.2 | 4.57 |
| C-1 phenanthrenes/anthracenes | P1 | 3 | 192.3 | 5.04 |
| C-2 phenanthrenes/anthracenes | P2 | 3 | 206.3 | 5.46 |
| C-3 phenanthrenes/anthracenes | P3 | 3 | 220.3 | 5.92 |
| C-4 phenanthrenes/anthracenes | P4 | 3 | 234.3 | 6.32 |
| anthracene | ANT | 3 | 178.2 | 4.53 |
| fluoranthene | FLU | 3 | 202.3 | 5.08 |
| pyrene | PYR | 3 | 202.3 | 4.92 |
| C-1 fluoranthenes/pyrenes | FP1 | 3 | 216.3 | 5.48 |
| C-2 fluoranthenes/pyrenes | FP2 | 3 | 230.3 | 6.15 |
| C-3 fluoranthenes/pyrenes | FP3 | 3 | 244.3 | 6.60 |
| C-4 fluoranthenes/pyrenes | FP4 | 3 | 258.3 | *7.22 |
| benzo(a)anthracene | BAA | 4 | 228.3 | 5.89 |
| chrysene | C0 | 4 | 228.3 | 5.71 |
| C-1 chrysenes | C1 | 4 | 242.3 | 6.14 |
| C-2 chrysenes | C2 | 4 | 256.3 | 6.43 |
| C-3 chrysenes | C3 | 4 | 270.4 | 6.94 |
| C-4 chrysenes | C4 | 4 | 284.4 | 7.36 |
| benzo(b)fluoranthene | BBF | 6 | 252.3 | 6.27 |
| benzo(k)fluoranthene | BKF | 6 | 252.3 | 6.29 |
| Benzo(e)pyrene | BEP | 6 | 252.3 | 6.44 |
| Benzo(a)pyrene | BAP | 6 | 252.3 | 6.11 |
| Perylene | PER | 5 | 252.3 | 6.44 |
| indeno(1,2,3-cd)pyrene | ICP | 6 | 276.3 | 6.72 |
| dibenzo(a,h)anthracene | DBA | 6 | 278.4 | 6.71 |
| benzo(ghi)perylene | BZP | 6 | 276.3 | 6.51 |

Deuterated surrogates were naphthalene-d_8_ (1), acenaphthene-d_10_ (2), phenanthrene-d_10_ (3), chrysene-d_12_ (4), perylene-d_12_ (5), and benzo[*a*]pyrene-d_12_ (6). Asterisks indicate interpolated log *K_ow_* estimates.
