# Supplementary material for: Spilled Oils: Static Mixtures or Dynamic Weathering and Bioavailability?
Source: PLoS One. 2015 Sep 2;10(9):e0134448. doi: 10.1371/journal.pone.0134448 (PMC4557949; doi:10.1371/journal.pone.0134448)
Supplement: S4 Table — (DOCX) [file pone.0134448.s008.docx]

**S4 Table.**

| **Abbreviation** | **Alkane** |
| --- | --- |
| C9alk | n-nonane |
| C10alk | n-decane |
| C11alk | n-undecane |
| C12alk | n-dodecane |
| C13alk | n-tridecane |
| C14alk | n-tetradecane |
| C15alk | n-pentadecane |
| C16alk | n-hexadecane |
| C17alk | n-heptadecane |
| Prist | 2,6,10,14-tetramethylpentadecane (pristane) |
| C18alk | n-octadecane |
| Phyt | 2,6,10,14-tetramethylhexadecane (phytane) |
| C19alk | n-nonadecane |
| C20alk | n-eicosane |
| C21alk | n-heneicosane |
| C22alk | n-docosasne |
| C23alk | n-tricosane |
| C24alk | n-tetracosine |
| C25alk | n-pentacosane |
| C26alk | n-hexacosane |
| C27alk | n-heptacosane |
| C28alk | n-octacosane |
| C29alk | n-nonacosane |
| C30alk | n-triacontane |
| C31alk | n-hentriacontane |
| C32alk | n-dotriacontane |
| C33alk | n-tritriacontane |
| C34alk | n-tetratriacontane |
| C35alk | n-pentatriacontane |
| C36alk | n-hexatriacontane |

Pristane and phytane are branched isoprenoids; all others are straight-chain compounds.
