# Supplementary material for: Spilled Oils: Static Mixtures or Dynamic Weathering and Bioavailability?
Source: PLoS One. 2015 Sep 2;10(9):e0134448. doi: 10.1371/journal.pone.0134448 (PMC4557949; doi:10.1371/journal.pone.0134448)
Supplement: S5 Table — (DOCX) [file pone.0134448.s009.docx]

**S5 Table.**

| **Abbreviation** | **Biomarker: Isoprenoids** | **Target Ions** |
| --- | --- | --- |
| norprist | norpristane | 57 |
| prist | 2,6,10,14-tetramethylpentadecane (pristane) | 57 |
| phyt | 2,6,10,14-tetramethylhexadecane (phytane) | 57 |
|  |  |  |
|  |  |  |
| **Abbreviation** | **Biomarker: Triterpanes** | **Target Ions** |
| TR23 | C23 tricyclic terpane | 191 |
| TR24 | C24 tricycilic terpane | 191 |
| TR25a | C25 tricyclic terpane (a) | 191 |
| TR25b | C25 tricyclic terpane (b) | 191 |
| TET24 | C24 tetracyclic terpane | 191 |
| TR26a | C26 tricyclic terpane (a) | 191 |
| TR26b | C26 tricyclic terpane (b) | 191 |
| TR28a | C28 tricyclic terpane (a) | 191 |
| TR28b | C28 tricyclic terpane (b) | 191 |
| TR29a | C29 tricyclic terpane (a) | 191 |
| TR29b | C29 tricyclic terpane (b) | 191 |

| **Abbreviation** | **Biomarker: hopanes** | **Target Ions** |
| --- | --- | --- |
| Ts | 18α(H),21β(H)-22,29,30-trisnorhopane | 191 |
| Tm | 17α(H),21β(H)-22,29,30-trisnorhopane | 191 |
| H28 | 17α(H),18α(H),21β(H)-28,30-bisnorhopane | 191 |
| NOR25H | 17α(H),21β(H)-25-norhopane | 191 |
| H29 | 17α(H),21β(H)-30-norhopane | 191 |
| C29Ts | 18α(H),21β(H)-30-norneohopane | 191 |
| M29 | 17α(H),21β(H)-30-norhopane (normoretane) | 191 |
| OL | 18α(H) and 18β(H)-oleanane | 191 |
| H30 | 17α(H),21β(H)-hopane | 191 |
| NOR30H | 17α(H)-30-nor-29-homohopane | 191 |
| M30 | 17β(H),21α(H)-hopane (moretane) | 191 |
| H31S | 22S-17α(H),21β(H)-30-homohopane | 191 |
| H31R | 22R-17α(H),21β(H)-30-homohopane | 191 |
| GAM | Gammacerane | 191 |
| H32S | 22S-17α(H),21β(H)-30,31-bishomohopane | 191 |
| H32R | 22R-17α(H),21β(H)-30,31-bishomohopane | 191 |
| H33S | 22S-17α(H),21β(H)-30,31,32-trishomohopane | 191 |
| H33R | 22R-17α(H),21β(H)-30,31,32-trishomohopane | 191 |
| H34S | 22S-17α(H),21β(H)-30,31,32,33-tetrakishomohopane | 191 |
| H34R | 22R-17α(H),21β(H)-30,31,32,33-tetrakishomohopane | 191 |
| H35S | 22S-17α(H),21β(H)-30,31,32,33,34-pentakishomohopane | 191 |
| H35R | 22R-17α(H),21β(H)-30,31,32,33,34-pentakishomohopane | 191 |

| **Abbreviation** | **Biomarker: steranes** | **Target Ions** |
| --- | --- | --- |
| S22 | C_22_ 5α(H),14β(H),17β(H)-sterane | 217,218 |
| DIA27S | C_27_ 20S-13β(H),17α(H)-diasterane | 217,218 |
| DIA27R | C_27_ 20R-13β(H),17α(H)-diasterane | 217,218 |
| C27S | C_27_ 20S-5α(H),14α(H),17α(H)-cholestane | 217,218 |
| C27BBR | C_27_ 20R-5α(H),14β(H),17β(H)-cholestane | 217,218 |
| C27BBS | C_27_ 20S-5α(H),14β(H),17β(H)-cholestane | 217,218 |
| C27R | C_27_ 20R-5α(H),14α(H),17α(H)-cholestane | 217,218 |
| C28S | C_28_ 20S-5α(H),14α(H),17α(H)-ergostane | 217,218 |
| C28BBR | C_28_ 20R-5α(H),14β(H),17β(H)-ergostane | 217,218 |
| C28BBS | C_28_ 20S-5α(H),14β(H),17β(H)-ergostane | 217,218 |
| C28R | C_28_ 20R-5α(H),14α(H),17α(H)-ergostane | 217,218 |
| C29S | C_29_ 20S-5α(H),14α(H),17α(H)-stigmastane | 217,218 |
| C29BBR | C_29_ 20R-5α(H),14β(H),17β(H)-stigmastane | 217,218 |
| C29BBS | C_29_ 20S-5α(H),14β(H),17β(H)-stigmastane | 217,218 |
| C29R | C_29_ 20R-5α(H),14α(H),17α(H)-stigmastane | 217,218 |
