# Supplementary material for: Spilled Oils: Static Mixtures or Dynamic Weathering and Bioavailability?
Source: PLoS One. 2015 Sep 2;10(9):e0134448. doi: 10.1371/journal.pone.0134448 (PMC4557949; doi:10.1371/journal.pone.0134448)
Supplement: S6 Table — (DOCX) [file pone.0134448.s010.docx]

**S6 Table.**

| **Name** | **type** | **pPAH** | **pAlk** | **pBio** | **ΣD/ΣP** | **w** | **pNaph** | **pChrys** | **pHeavyAlk** |
| --- | --- | --- | --- | --- | --- | --- | --- | --- | --- |
| PST-11-C | IFO_H_ | 0.27 | 0.71 | 0.019 | 0.94 | 1.17 | 30.69 | 4.31 | 15.32 |
| PST-11-C | IFO_H_ | 0.28 | 0.70 | 0.019 | 0.92 | 1.18 | 30.69 | 4.38 | 13.84 |
|  |  |  |  |  |  |  |  |  |  |
| PST-5-F | IFO_L_ | 0.58 | 0.41 | 0.003 | 0.38 | -0.71 | 51.51 | 1.56 | 2.56 |
| PST-5-F | IFO_L_ | 0.61 | 0.39 | 0.003 | 0.36 | -0.71 | 51.86 | 1.54 | 2.39 |
| PST-9-A | IFO_L_ | 0.59 | 0.41 | 0.003 | 0.35 | -0.82 | 52.02 | 1.49 | 2.49 |
| PST-9-A | IFO_L_ | 0.59 | 0.41 | 0.003 | 0.34 | -0.82 | 51.55 | 1.47 | 2.29 |
| PST-9-A | IFO_L_ |  |  |  | 0.34 | -0.82 | 51.63 | 1.49 |  |
| #4 Port IFO | IFO_L_ | 0.58 | 0.42 | 0.003 | 0.36 | -0.68 | 51.06 | 1.58 | 2.76 |
| #4 Port IFO | IFO_L_ | 0.60 | 0.39 | 0.003 | 0.35 | -0.68 | 51.31 | 1.55 | 2.64 |
| #4 Port IFO | IFO_L_ |  |  |  | 0.35 | -0.68 | 51.29 | 1.58 |  |
|  |  |  |  |  |  |  |  |  |  |
| PST-8-A | MDO |  |  |  | 0.86 | -3.08 | 62.00 | 0.47 |  |
| PST-8-A | MDO | 0.21 | 0.79 | 0.000 | 0.71 | -3.08 | 62.71 | 0.44 | 2.21 |

Proportionate composition of polynuclear aromatic hydrocarbons pPAH, alkanes (pAlk), and biomarkers (pBio) are tabulated. Proportionate dibenzothiophene content relative to phenanthrenes (ΣD/ΣP) distinguishes IFO_H_ from IFO_L_. Weathering (w, estimated by modeling; Short and Heintz 1997) summarizes PAH composition; w is least where proportions of naphthalenes (pNaph) are greatest and proportions of chrysenes (pChrys) are least. Weathering is also reflected in proportions of heavy alkanes (≥C27) relative to total alkanes. Weathering in this context reports source oil condition and does not imply environmental change.
